# High-Precision Centroid Measurement Method Based on 3D Scanning and Hooke’s Law

**DOI:** 10.3390/s25237210

**Published:** 2025-11-26

**Authors:** Xin He, Zhen Li, Xin Pan, Yong Yang

**Affiliations:** 1 Engineering Training Center, Tianjin University of Technology and Education, Tianjin 300222, China; 2 School of Mechanical Engineer, Tianjin University of Technology and Education, Tianjin 300222, China; 3 Institute of Flexible Electronics Technology of THU, Jiaxing 314000, China

**Keywords:** centroid measurement, multi-point weighing method, 3D scanning, Hooke’s law

## Abstract

The accurate determination of an object’s centroid is a critical requirement in fields such as aerospace engineering and advanced manufacturing, where it is essential for quality control and system performance. Traditional methods, such as multi-point weighing, are often limited by restricted measurement ranges, inaccuracies from mechanical alignment tolerances, and susceptibility to lateral force interference from uneven platforms, which collectively constrain measurement precision. To address these challenges, a novel measurement framework is proposed that synergizes high-precision 3D scanning with Hooke’s law-based mechanical sensing. This methodology eliminates dependencies on mechanical positioning and offers enhanced compatibility with various object geometries through its non-contact 3D scanning. The system also integrates linear spring-based force transduction for enhanced load adaptability and incorporates active anti-tilt compensation using 3D scanning and motor leveling. Experimental validation demonstrated sub-millimeter accuracy compared to the multi-point weighing method, with measured centroid deviations of 0.01 mm (*X*-axis), 0.06 mm (*Y*-axis), and 0.03 mm (*Z*-axis), achieving a composite spatial precision of 0.07 mm. This methodological innovation not only expands the operational envelope of centroid measurement systems but also provides new theoretical insights and a robust methodology for measuring complex parts and systems.

## 1. Introduction

Centroid measurement plays a critical role in numerous engineering and scientific applications, particularly in aerospace engineering, precision manufacturing, robotics, and inertial system calibration [1,2,3]. Accurate determination of an object’s mass center directly impacts dynamic balance, structural stability, and control accuracy. For instance, in satellite attitude control systems, even sub-millimeter centroid deviations can lead to significant orientation errors over long durations [4]. Similarly, in precision machining and additive manufacturing, component imbalances caused by centroid misalignments can degrade machining accuracy and lead to premature tool wear [5]. In the aerospace field, the accurate measurement of centroid has even broader significance. For spacecraft, precise centroid determination is essential during both ground testing and in-orbit operations, as it directly affects attitude stability, fuel consumption efficiency, and orbit maintenance. A minor offset in the centroid of a satellite or launch vehicle can cause cumulative deviations in attitude control, which not only increases the demand for attitude correction but also accelerates the consumption of limited propellant resources, ultimately reducing mission lifespan. Moreover, for high-speed aircraft, unmanned aerial vehicles (UAVs), and re-entry vehicles, small centroid misalignments can induce aeroelastic instabilities and adversely impact flight safety. Accurate centroid localization is also vital for payload integration in multi-satellite missions or deep-space probes, where unbalanced mass distributions could lead to structural vibrations and impaired sensor measurements. Therefore, achieving high-precision centroid measurement is not only a fundamental requirement for structural design and dynamic testing but also a key enabler for enhancing the reliability, efficiency, and longevity of aerospace systems.

The most commonly used method for centroid measurement in practice is the multi-point weighing method, wherein the object is weighed in different orientations to deduce the centroid using moment equilibrium equations [6]. This approach is straightforward and can be realized using commercially available force sensors or weighing platforms. However, it has several inherent limitations. First, the measurement range is constrained by the physical dimensions of the weighing apparatus and the need for repositioning the object [7]. Large or irregularly shaped components become challenging to measure accurately. Second, mechanical alignment tolerances and positioning errors during rotation or flipping introduce significant inaccuracies, especially in the lateral directions [8]. Third, external disturbances, such as platform tilt, local friction, or uneven supporting surfaces, result in lateral force components that distort the vertical force readings, further compromising precision [9].

The multi-point weighing method, which employs three or more precision load cells supporting the test specimen, has emerged as a leading static technique for simultaneously measuring mass and the center of gravity of rigid bodies in high accuracy applications [10]. Wang et al. proposed a structured four-sensor measurement system that significantly reduces lateral force interference by introducing a flexible load-bearing design [11]. This configuration achieved high repeatability, with mass error less than 0.05% and centroid error within ±0.3 mm, demonstrating its reliability for general-purpose rigid body measurements. Zhang et al. developed a flexible measurement system capable of accommodating large and irregular geometries [12]. By utilizing two independent weighing stations and aligning their coordinate systems, they realized centroid measurement accuracy better than 1 mm. To address the needs of aerial robotics, Sun et al. tailored the multi-point weighing method for lightweight unmanned aerial vehicle (UAV) platforms, introducing a three-point static weighing setup combined with a 3D analytical model of centroid coordinates [13]. Their work also included a comprehensive error sensitivity analysis, providing a valuable reference for miniature systems. In the aerospace domain, Zhang et al. introduced a generalized weighing platform for large aircraft components [14]. This platform integrates four high-precision force sensors with a modular base and a dynamic leveling system, enhancing measurement robustness under large loads. These studies collectively demonstrate that multi point weighing allows highly accurate centroid measurement with relatively simple hardware, and can be tailored to applications from small UAV components up to very large aerospace structures [15]. However, its limitations include sensitivity to side loading, strict requirements for sensor alignment and leveling, and reduced flexibility when measuring non rigid or non-planar geometries [16]. Such limitations have been addressed in advanced designs via flexible sensor arrangements and multi station error compensation, but further adaptation to complex and non-standard shapes remain a challenge.

To overcome these constraints, a high-precision centroid measurement framework is proposed, which integrates non-contact 3D scanning with Hooke’s law-based mechanical sensing. The key innovation lies in eliminating the need for mechanical repositioning by using 3D scanning to obtain a precise point cloud model of the object’s external geometry in a single setup. From this, the geometric centroid is computed directly. Simultaneously, the object’s gravitational force is measured via linear spring-based deformation sensors, whose response is governed by Hooke’s law. This approach provides high sensitivity while ensuring structural simplicity and mechanical robustness. To further mitigate tilt-induced errors, a leveling system based on motorized adjustment from the 3D scan data is introduced, enabling platform leveling and compensation of lateral forces. This study contributes to the broader field of metrological innovation by demonstrating a new paradigm of geometry-driven, force-coupled centroid measurement, expanding the applicability of centroid localization techniques to a wider class of components and systems. It offers both practical value for industrial quality control and theoretical insight into multi-domain measurement integration.

## 2. Materials and Methods

### 2.1. Overall Design of Centroid Measurement System

**Figure 1:**
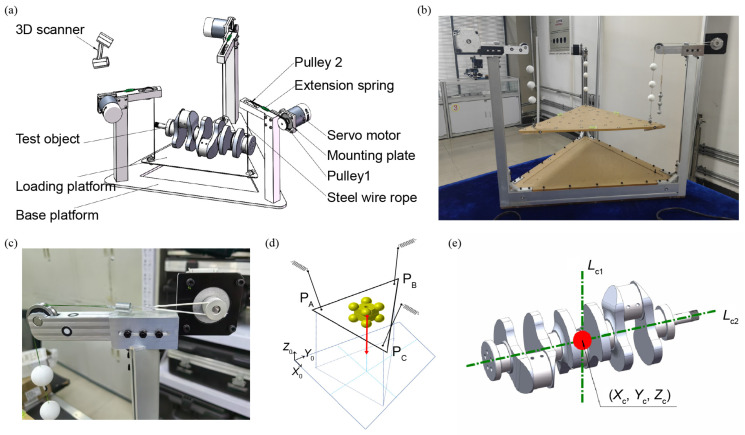
Overview of the centroid measurement system based on 3D scanning and Hooke’s law. (**a**) Schematic illustration of the overall structure. (**b**) The appearance of the centroid measurement system. (**c**) The spring is used as a force sensitive element to obtain the measured force based on the position of the feature sphere based on Hooke’s law. (**d**) Schematic diagram of the principle of centroid measurement based on 3D scanning and Hooke’s law. (**e**) Schematic diagram of determining the centroid using multiple centroid lines.

As illustrated in Figure 1a, the proposed centroid measurement system consists of a base platform supporting three vertical suspension rods, each equipped with a horizontally mounted servo motor, a calibrated extension spring (galvanized steel, 1 mm × 10 mm × 48 mm), and a guide pulley. Three steel wire ropes (304 stainless steel, with a diameter of 0.4 mm and a tensile strength of 10 kg) are routed from each motor through the respective spring and guide pulley, and terminate at feature spheres (Polyvinyl chloride, 25 mm diameter, 0.4 mm through hole) before connecting to a loading platform. This configuration allows the platform to be suspended via three ropes, forming a triangulated support system with leveling capability for anti-tilt compensation [see Figure 1b]. As illustrated in Figure 1c, the servo motors adjust the effective length of each wire rope, enabling fine-tuned leveling of the loading platform. The stepper motor has a step angle of 0.9° with a positioning uncertainty of approximately ±0.02 mm at the output shaft, which ensures fine control of movement and positioning during the experiment. A mechanical bubble level is mounted on the platform to provide visual feedback, ensuring that the test object remains approximately horizontal during operation to eliminate lateral force interference. Its sensitivity is 0.02 mm/m, which corresponds to an angular resolution of about 20 μrad, providing sufficient sensitivity for detecting very small inclinations. The mechanical principal diagram of the system, i.e., the improved multi-point weighing measurement principle, is shown in Figure 1d.

A high-precision handheld 3D scanner (Creaform HandySCAN 700, Creaform Inc., Lévis, QC, Canada) is employed to acquire full point cloud data of the measurement system. This scanner offers a resolution of 0.05 mm, an accuracy of 0.03 mm, and a volumetric accuracy of 0.02 mm + 0.06 mm/m, with a scanning window of 275 mm × 250 mm. During measurement, the scanner captures the spatial positions of distributed reference points affixed to key components, including the feature spheres at the ends of the wire ropes. This allows for a full 3D reconstruction of the system, including the change in position in the feature sphere reflecting the deformation of the extension spring. By transforming the pose of the object to be measured, multiple centroid lines are obtained, and the centroid coordinates can be measured by solving the intersection point, as shown in Figure 1e. The measured object should have sufficient structural rigidity to prevent deformation during placement and a uniform, non-reflective surface suitable for optical scanning. The mass of the object is limited to the linear elastic range of the supporting springs to ensure accurate force measurement and linear response according to Hooke’s law.

This design confers several advantages. First, the reliance on traditional mechanical alignment and rigid fixture tolerances is eliminated by leveraging 3D scanning for structural referencing, thereby mitigating common positioning errors. Second, the non-contact nature of scanning enhances compatibility with various object geometries. Third, by measuring the vertical displacement of the feature spheres relative to the fixed endpoints of each spring, the system captures the elongation of each spring under load. Each spring is pre-calibrated to determine its stiffness coefficient, enabling force measurement via Hooke’s law. By selecting springs of appropriate stiffness ranges, the system can be easily adapted to accommodate a wide spectrum of object weights, from light components to large aerospace structures. Combining these measured forces with the geometric information provided by 3D scanning, the centroid of the object placed on the platform is computed using a modified multi-point weighing algorithm. This hybrid approach enables high-precision, robust centroid determination without the need for complex mechanical setups or frequent repositioning.

### 2.2. Modeling the Centroid Measurement System

**Figure 2:**
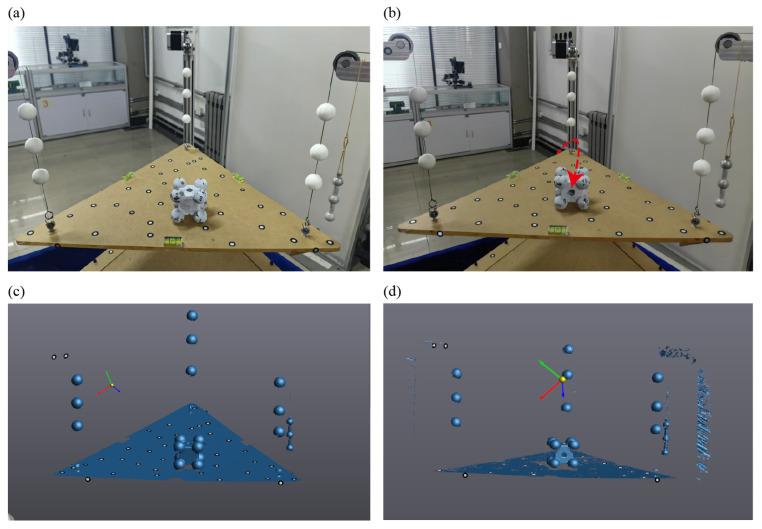
Schematic diagram of the centroid measurement process. (**a**) The standard component to be tested is placed on the loading platform of the system. (**b**) The placement orientation of the standard component is changed, with a red dashed arrow indicating the rotation direction. The through-hole orientation clearly differs before and after rotation. (**c**) Three-dimensional scanning of the standard component in its initial orientation. (**d**) Three-dimensional scanning of the standard component in a transformed orientation.

Before the measurement, the loading platform should be leveled by adjusting Pulley 1 using the motor. This is determined by checking the bubble level on the platform. The object to be measured is then placed at the center of the platform, as shown in Figure 2a. If the platform remains significantly tilted, it can be approximately leveled again using the motor (perfect leveling is not required). Subsequently, 3D modeling of both the centroid measurement system and the object using a scanner. The 3D point cloud data of the test object, the platform, and feature spheres are captured by the scanner [see Figure 2c]. They are processed to calculate the object’s centroid based on the improved multipoint weighing method, as illustrated in Figure 1d. A coordinate system is established such that its *Z*-axis is aligned perpendicularly to the loading platform surface. Considering an object of weight *W* (mass *m*, *W* = *mg*) to be measured supported by three vertical linear springs whose contact points in the system reference plane have coordinates $\left(x_{i},y_{i}\right)$ for i=1,…,n. Let the vertical reaction force at the i-th support be $F_{i}$. Each spring obeys Hooke’s law $F_{i}=k_{i}z_{i}$, where $k_{i}$ and $z_{i}$ are the stiffness and measured displacement of the i-th spring. We take the origin and axes of the coordinate system such that the gravity direction is −*z*, and *x*, *y* span the horizontal support plane. The centroid of the object projects onto the support plane at $\left(\bar{x},\bar{y}\right)$. Static equilibrium requires that the sum of vertical reactions equals the weight,

(1) $$\sum_{i=1}^{n}F_{i}=W.$$

For moments about the *y*-axis (i.e., taking moments that give the first moment about y and relate to *x*-coordinate of the resultant), the moment due to the weight *W* acting at $\bar{x}$ must be balanced by the moments of the support forces. An analogous condition holds for moments about the *x*-axis, leading to the relation for $\bar{y}$.

(2) $$\left\{\begin{array}{c} \sum_{i=1}^{n}F_{i}x_{i}=W\cdot \bar{x}.\\ \sum_{i=1}^{n}F_{i}y_{i}=W\cdot \bar{y}. \end{array}\right.$$

Based on Hooke’s law, the deformation length of the springs can be determined from the center positions of the feature spheres, allowing calculation of the centroid projection on the *XY* plane,

(3) $$\left\{\begin{array}{l} \bar{x}=\frac{x_{1}\cdot k_{1}z_{1}+x_{2}\cdot k_{2}z_{2}+x_{3}\cdot k_{3}z_{3}}{k_{1}z_{1}+k_{2}z_{2}+k_{3}z_{3}}\\ \bar{y}=\frac{y_{1}\cdot k_{1}z_{1}+y_{2}\cdot k_{2}z_{2}+y_{3}\cdot k_{3}z_{3}}{k_{1}z_{1}+k_{2}z_{2}+k_{3}z_{3}} \end{array}\right.,$$

It is derived from the principle of static equilibrium, where the centroid coordinates are determined based on the torque balance of the object under gravity. The derivation is based on several key assumptions: the test object behaves as a rigid body without deformation during measurement; each support point provides only a vertical reaction force and remains stationary; the mass distribution of the object stays constant during rotation or repositioning; gravitational acceleration is uniform and constant; and the coordinate system of the supporting plane is fixed and aligned with the measurement reference frame. Under these conditions, Equation (3) accurately expresses the centroid coordinates as a function of the measured forces and the geometric positions of the support points. According to Hooke’s law, the elastic deformation of each supporting spring is linearly proportional to the applied load. By measuring the displacement of each spring under static equilibrium, the corresponding supporting force can be calculated. These forces reflect the distribution of the object’s gravitational load on each support point. The centroid projection on the supporting plane can then be derived from the equilibrium of moments among these forces. Based on Hooke’s law, the tensile force in each steel wire can be calculated. Once the projection of the centroid onto the *XY* plane is obtained, a line parallel to the *Z*-axis can be drawn through this point to determine the centroid line *l_i_* under the current measurement condition. Then, the orientation of the object on the platform is changed to obtain a new centroid line corresponding to this new state, as shown in Figure 2b.

(4) $$P_{N}=R\cdot P_{0}+P_{s}$$

where *P_s_* and *R* can be obtained from the object’s rotation and translation, as measured by the scanner. *P*_0_ represents the centroid coordinate in the original coordinate system, and *P*_N_ represents the centroid coordinate in the new coordinate system. Three-dimensional scanning of the standard component in a transformed orientation is shown in Figure 2d. After acquiring two or more centroid lines of the test object, these lines are transformed into a common coordinate system based on the target point cloud data. By repeating the measurement under different orientations, multiple centroid projection lines are obtained, and their intersection determines the three-dimensional centroid coordinates. Taking the theoretical model of the object as the reference, both the point cloud and the centroid lines from each measurement are transformed into the coordinate system of the theoretical model. Through these coordinate transformations, the equation of the centroid *P*_01_ in the original coordinate system can be back-calculated,

(5) $$P_{01}=R^{-1}(P_{N}-P_{s}),$$

The centroid lines obtained from multiple measurements are transformed into a common coordinate system. Then, the least squares method is applied to determine the optimal intersection point of these lines, which corresponds to the centroid coordinates of the measured object.

## 3. Results

### 3.1. Accuracy Test with Standard Parts

**Figure 3:**
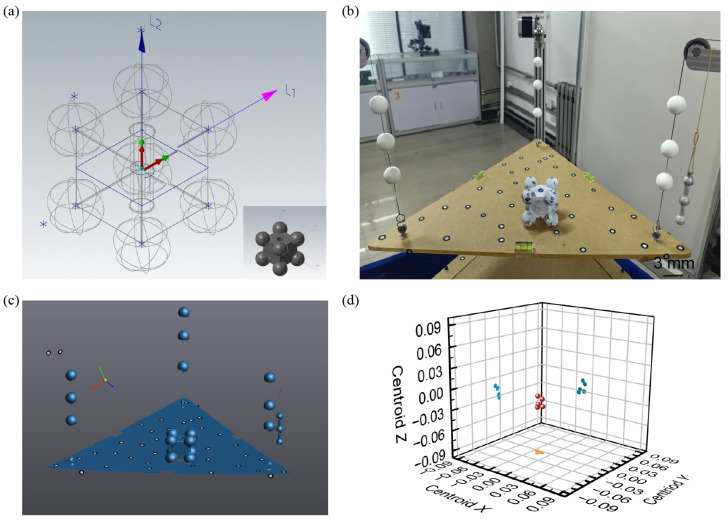
Accuracy test of the centroid measurement system using the standard component. (**a**) Schematic diagram of the standard component. The theoretical centroid is calculated based on its ideal geometry and material properties. Inset: 3D model of the standard component. (**b**) Photograph of the standard component used for system accuracy testing. (**c**) Three-dimensional scanning of the standard component used for system accuracy testing. (**d**) The results of the five centroid point measurements demonstrate good measurement repeatability.

As shown in Figure 3a, the standard component is composed of eight spheres embedded at the corners of a cube (cube dimensions: 40 × 40 × 40 mm^3^; sphere diameter: 25 mm), with a central through-hole of 10 mm diameter in the rectangular body. The component is made of a single material (45# steel), so its theoretical centroid can be calculated directly from the 3D scanned model, serving as a reference to validate the accuracy of the measurement system. Figure 3b shows a photograph of the system during the centroid testing of the standard component. The standard block was placed at arbitrary orientations for three independent measurements. The point cloud data obtained from these measurements were then analyzed to extract the centroid lines [see Figure 3c]. A plane was fitted to the sample platform and aligned with the *XY* plane of the global coordinate system, transforming the entire point cloud into this global frame. The *Z*-axis direction corresponds to the orientation of the centroid lines. Combined with the spring deformation data, the *X* and *Y* coordinates of the centroid line projection were calculated using Equation (1), thereby constructing the full centroid line. Finally, both the point cloud data and the centroid lines of the test object were aligned with the theoretical model.

Based on the theoretical model and its centroid coordinates, the measurement error can be evaluated. The results from five measurements are presented in Table 1. The theoretical coordinates of the centroid point *G* of the standard component are determined using 3D software to be (0, 0, 0). After scanning, each fitted centroid line was parameterized using two 3D points. In the first measurement, the centroid lines of the standard component were defined by the following coordinate pairs. Line *L*_1_ extended from (−0.097, 0.020, −50) to (−0.054, −0.030, 50), while Line *L*_2_ extended from (0.039, 50, −0.070) to (0.012, −50, −0.060). The intersection point of these two centroid lines was calculated using the least squares method, yielding the measured centroid point *T*. Averaging the results across five repeated experiments gave a final measured centroid of *T* = (−0.019, −0.004, −0.024) [see Figure 3d]. These results indicate that the prototype exhibits coordinate errors of −0.019 mm in the *x*-direction, −0.004 mm in the *y*-direction, and −0.024 mm in the *z*-direction, thereby confirming the measurement accuracy of the system.

### 3.2. Measurement System Validation on Representative Models

**Figure 4:**
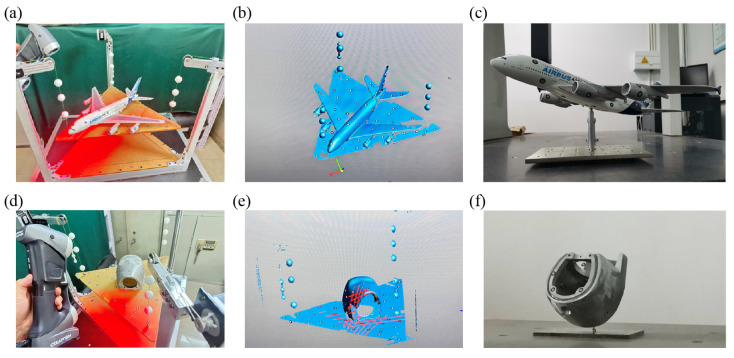
Centroid measurements on two physical models, a scaled aircraft model and a human skull model. (**a**) Experimental photograph, (**b**) corresponding 3D scanning result, and (**c**) validation photograph where the scaled aircraft model is supported at the measured centroid position. (**d**) Experimental photographs, (**e**) corresponding 3D scanning result, and (**f**) validation photograph of the human skull model.

To further validate the performance and applicability of the proposed centroid measurement system, experiments were conducted on two representative physical models—a scaled aircraft model and a human skull model. These models were chosen to represent complex geometries and heterogeneous mass distributions, which are commonly encountered in engineering and biomedical applications. Each model was placed on the measurement system in arbitrary orientations, and five independent measurements were performed for each to ensure repeatability. The final centroid coordinates were obtained by averaging the results from these repeated measurements. Figure 4a–c show the scaled aircraft model: (a) experimental photograph, (b) the corresponding 3D scanned point cloud, and (c) validation photograph where the model is supported precisely at the measured centroid. Figure 4d–f show the human skull model: (d) experimental photograph, (e) the corresponding 3D scanned point cloud, and (f) validation photograph with the model balanced at the identified centroid. The successful balance of both models at their measured centroid positions confirms the accuracy, repeatability, and broad applicability of the proposed system, demonstrating its effectiveness for both engineering and biomedical applications.

### 3.3. Error Analysis of Centroid Measurement

#### 3.3.1. Measurement Error of 3D Scanner

The spatial accuracy of the HandySCAN 700 (0.02 mm + 0.06 mm/m) introduces a small uncertainty in determining the positions of the reference spheres and structural features. Hence, the scanner-induced positional uncertainty depends on the measurement scale: for the standard component used here (max dimension 40 mm) the uncertainty is 0.022 mm.

#### 3.3.2. Sphere Center Localization Error

The centers of the three reference spheres were determined by least-squares fitting of the scanned point cloud data. Considering the scanner’s point accuracy and the sampling density, the theoretical localization uncertainty of the fitted centers was estimated to be less than 0.001 mm, and thus can be regarded as negligible.

#### 3.3.3. Measurement Error of Spring Stiffness

The stiffness coefficients of the testing springs were precisely calibrated using a high-accuracy force gauge (millinewton resolution) and a micro-positioning stage (micrometer resolution). The calibrated stiffness value for the springs was *K* = 2 N/mm. Owing to the high precision of this calibration procedure, the uncertainty in the stiffness constant was negligible and did not contribute significantly to the overall measurement error.

#### 3.3.4. Thermal Drift

The structural components of the measurement system were primarily made of steel, with a linear thermal expansion coefficient of approximately 10^−5^/°C. During the experiments, the ambient temperature variation was maintained within ±1 °C. Under these conditions, the maximum dimensional change caused by thermal expansion was estimated to be at the micrometer level. Therefore, the contribution of thermal expansion to the centroid determination uncertainty is negligible compared with the overall measurement accuracy.

#### 3.3.5. Alignment and Leveling Error

The rope angle deviation θ from the true vertical direction introduces a cosine error in the measured vertical force component, expressed as $F_{z}=Fcos\theta$. For small angular deviations, the relative error can be approximated as $1-cos\theta \approx \theta^{2}/2$. During the experiment, the leveling accuracy of the platform was controlled within 20 μrad (0.0011°), corresponding to a cosine error below $1\times 10^{-10}$, which is negligible compared with other uncertainty sources.

In addition to the platform leveling uncertainty, it should be noted that the reaction forces measured by the springs may not be perfectly vertical if the pulleys guiding the ropes are slightly misaligned with respect to the true vertical direction. Such misalignment introduces a small inclination angle of the rope, resulting in a minor cosine error in the measured force. For the current setup, considering typical machining and assembly errors, the pulley installation accuracy is estimated to be better than 0.5°, corresponding to a maximum force measurement deviation of less than 0.04% due to the cosine effect. Hence, the influence of this potential rope inclination on the final centroid determination is negligible.

#### 3.3.6. Evaluation of the Combined Uncertainty

Based on the root-sum-square propagation of the identified uncertainty sources, the combined standard uncertainty $u_{c}$ of the centroid measurement system was estimated to be approximately 0.022 mm. Considering a coverage factor of *k* = 2 (corresponding to a 95% confidence level), the expanded uncertainty U was determined to be 0.044 mm.

### 3.4. Centroid Measurement of Special-Shaped Mass Blocks

**Figure 5:**
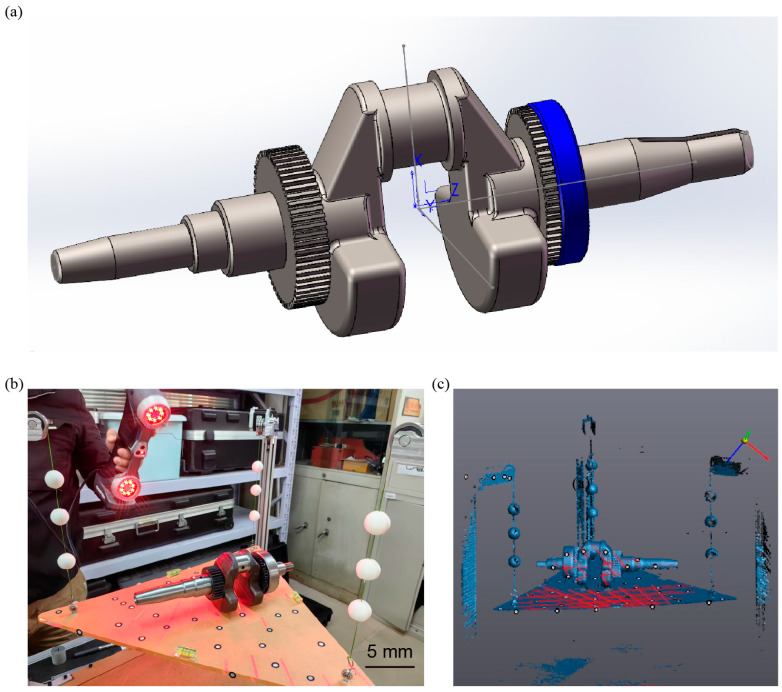

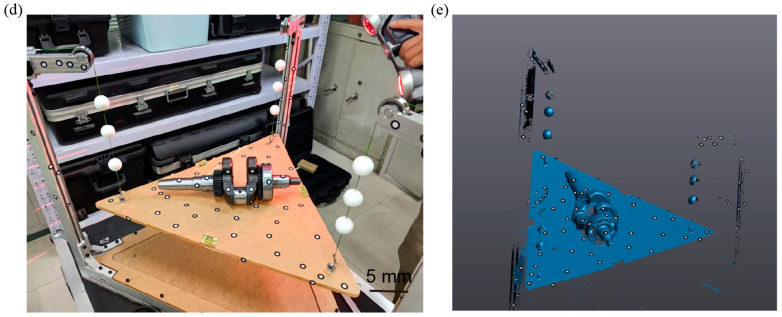
Centroid measurement of the crankshaft of an air-cooled diesel generator. (**a**) Schematic diagram of the crankshaft. (**b**) Three-dimensional scanning of the crankshaft and the centroid measurement system using a scanner. (**c**) The 3D scanning results provide the pose of the crankshaft and the spatial coordinates of the feature spheres. (**d**) Three-dimensional scanning of the crankshaft in transformed poses using a scanner. (**e**) The position of the crankshaft in transformed poses and the spatial coordinates of the feature spheres.

To further demonstrate the performance of the measurement system in determining the centroid of test objects, centroid measurement was carried out on the crankshaft of an air-cooled diesel generator, which has an irregular shape and is composed of multiple materials, as shown in Figure 5a. The crankshaft has a total length of 300 mm and a journal diameter of 38 mm. For test objects of this kind, the multipoint weighing method typically requires a dedicated testing platform and multiple customized fixtures to achieve high-precision centroid measurement. In our experiments, the crankshaft was measured in three different poses for the multi-point weighing procedure.

Using the proposed method, the centroid coordinates were obtained by averaging five independent measurements to ensure repeatability. Photographs of the crankshaft during the measurement process are shown in Figure 5b,d. Two 3D point cloud datasets were acquired under different pose conditions through scanning, resulting in two centroid lines, each defined by two 3D coordinate points [see Figure 5c,e]. The two centroid lines are line *L*_1_ extending from (5.59, −61.39, 0.46) to (5.59, 383.17, 0.46) and line *L*_2_ extending from (−91.95, 151.25, 5.64) to (88.93, 151.25, 5.64), so the centroid is (151.25, 0.46, 5.64). The centroid coordinate measured by multi-point weighing method is (151.24, 0.40, 5.61). The measured centroid point differed from that obtained by the multi-point weighing method by only 0.07 mm, confirming the accuracy of the proposed approach.

## 4. Discussion

In conclusion, a high-precision centroid measurement method that integrates 3D scanning and Hooke’s law-based mechanical sensing is proposed to address the inherent limitations of traditional multi-point weighing techniques. By leveraging non-contact 3D scanning for structural referencing and integrating spring deformation sensing for force quantification, the system eliminates the need for mechanical repositioning and enhances adaptability to complex and irregular geometries. The incorporation of motorized leveling further mitigates tilt-induced errors, enabling stable and repeatable measurements. Experimental validation using both a standard component and an irregular crankshaft with heterogeneous materials confirmed the system’s effectiveness. The measured centroid errors were within 0.07 mm compared to the multi-point weighing method, demonstrating excellent accuracy and repeatability. Notably, the system maintained its performance even for geometrically complex and heavy objects, where conventional methods would require customized fixtures and substantial setup effort.

This work contributes a novel, geometry-driven and force-coupled approach to centroid localization, offering both theoretical insight and practical tools for extending the capabilities of centroid measurement systems. The proposed method holds promise for a wide range of applications in aerospace, precision manufacturing, and structural calibration, particularly where traditional measurement techniques face constraints in precision, scalability, or compatibility. Future work will focus on expanding the system’s automation capabilities, integrating AI-based point cloud registration, and extending applicability to dynamic or in situ measurement scenarios.

## Figures and Tables

**Table 1:** Centroid lines and centroids of five replicate measurements.

Number	*L*_1_ (mm)	*L*_2_ (mm)	Centroid (mm)
1	(−0.097, 0.02, −50)	(0.039, 50, −0.07)	(−0.025, −0.002, −0.033)
	(−0.054, −0.03, 50)	(0.012, −50, −0.06)	
2	(−0.081, 0.02, −50)	(0.041, 50, −0.01)	(−0.018, −0.01, −0.013)
	(−0.042, −0.02, 50)	(0.010, −50, −0.04)	
3	(−0.085, 0.01, −50)	(0.025, 50, −0.05)	(−0.023, −0.003, −0.028)
	(−0.050, −0.02, 50)	(0.017, −50, −0.06)	
4	(−0.081, 0.02, −50)	(0.048, 50, −0.06)	(−0.015, −0.002, −0.03)
	(−0.031, −0.03, 50)	(0.004, −50, −0.06)	
5	(−0.078, 0.01, −50)	(0.039, 50, −0.05)	(−0.014, −0.005, −0.018)
	(−0.029, −0.03, 50)	(0.012, −50, −0.02)	

## Data Availability

All the data required to assess the conclusions are present in the main text. Additional information related to this paper may be requested from the authors.
